# Left colic artery–preserving radical rectal cancer surgery: a literature review

**DOI:** 10.3389/fsurg.2025.1644048

**Published:** 2026-01-05

**Authors:** Xiyin Yang, Yuanshui Sun, Qiang Hu

**Affiliations:** 1 Department of Traditional Chinese Medicine, Community Health Service Center of Guali Town of Xiaoshan, Hangzhou, China; 2 Department of Gastrointestinal Surgery, Tongde Hospital of Zhejiang Province, Hangzhou, China; 3 The Integrated Traditional Chinese and Western Medicine School of Clinical Medicine, Zhejiang Chinese Medicine University, Hangzhou, China; 4 Department of General Surgery, Tongde Hospital of Zhejiang Province, Hangzhou, China

**Keywords:** anastomotic leak, high-tie vs. low-tie ligation, indocyanine-green fluorescence perfusion, left colic artery preservation, rectal cancer surgery

## Abstract

**Objective:**

To review current evidence on left colic artery (LCA) preservation during radical rectal-cancer surgery, covering anatomic rationale, surgical techniques, oncologic safety and functional outcomes.

**Methods:**

PubMed, Embase, the Cochrane Library and CNKI were comprehensively searched for randomized trials, cohort studies and meta-analyses published in the past two decades that compared high-tie inferior mesenteric artery (IMA) ligation with low-tie LCA-preserving procedures. Key end-points included overall survival (OS), disease-free survival (DFS), local recurrence, anastomotic leak, urogenital and bowel function, and peri-operative recovery.

**Results:**

Across the aggregated literature, five-year OS, DFS, local-recurrence rates and R0 resection rates did not differ significantly between the two ligation strategies. Preserving the LCA consistently reduced clinical anastomotic leaks and was associated with better postoperative urinary, sexual and bowel function, as well as marginally shorter hospital stay and time to gastrointestinal recovery. Pre-operative CTA or MRA accurately delineates IMA-LCA branching patterns and collateral integrity, while intra-operative indocyanine-green fluorescence provides real-time perfusion assessment; emerging artificial-intelligence models show promise in refining both pre-operative planning and intra-operative decision-making.

**Conclusions:**

When meticulous D3 lymph-node clearance is maintained, LCA preservation delivers oncologic outcomes equivalent to high-tie ligation while offering notable advantages in peri-operative safety and long-term function. Consequently, low-tie LCA-preserving resection should be considered the preferred approach for most low-anterior resections unless specific anatomic or oncologic contraindications exist. Future multicentre randomised trials and AI-assisted studies are warranted to identify high-risk subgroups and optimise patient-specific indications.

## 1 Introduction

How best to manage the IMA during radical rectal cancer surgery has been debated for more than a century. As early as 1908, Moynihan and Miles described the two strategies still used today: high tie—ligation of the IMA at its origin from the aorta—and low tie—division distal to the LCA branch (1, 2). High tie permits complete lymph-node clearance at the IMA root and can yield a longer colonic segment for a low anastomosis, but sacrificing the LCA may reduce anastomotic perfusion and increase the risk of autonomic-nerve injury (3, 4). In contrast, low tie preserves the LCA and maintains arterial inflow to the descending and sigmoid colon, which may lower the incidence of anastomotic leak and better protect pelvic autonomic function, at the expense of a technically more demanding dissection and potentially limited bowel length (5, 6). Although high tie was historically assumed to improve oncological radicality, studies since the 1980s have not demonstrated a consistent survival advantage. emain mixed, making the choice of ligation level a hot topic in colorectal surgery (7).

With growing emphasis on local-recurrence patterns and long-term quality of life, LCA-preserving low-tie ligation has attracted renewed interest. Recent retrospective cohorts, prospective trials and meta-analyses have compared high- and low-tie strategies, yet their conclusions remain heterogeneous, and the optimal ligation level continues to be a key point of controversy in colorectal surgery.

This work is a narrative literature review based on a structured search of PubMed, Embase, the Cochrane Library and CNKI. We focused on randomized controlled trials, cohort studies and meta-analyses published between January 2000 and June 2025 that compared high-tie IMA ligation with low-tie LCA-preserving procedures in radical sigmoid or rectal cancer surgery. The main search terms included combinations of “inferior mesenteric artery ligation”, “left colic artery preservation”, “high tie”, “low tie”, “rectal cancer” and “sigmoid colon cancer”. we did not perform a formal quantitative meta-analysis or risk-of-bias assessment (e.g., ROBIS, AMSTAR); instead, we synthesised the evidence qualitatively, emphasising areas of agreement, controversy and implications for practice. We synthesize the latest evidence on anatomical rationale, operative technique, indications, oncological and functional outcomes of rectal-cancer resection with LCA preservation, and discuss current controversies and future research directions.

## 2 Anatomical and physiological foundations

### 2.1 Branching variations of the IMA

The IMA typically gives rise to three main branches: the LCA, sigmoidal arteries and the superior rectal artery, but marked anatomical variability is common. In a 3D CT angiography study of 471 patients, Murono et al. found that the LCA originated as an independent branch from the IMA trunk in 41.2% of cases, shared a common trunk with the sigmoidal arteries in 44.7%, and was congenitally absent in roughly 5.1%  (3). A separate angiographic series in older adults reported a similar 5 % absence rate, underscoring that a small subset of patients simply lacks a preservable LCA (4).

When present, the LCA supplies the descending and (proximal) sigmoid colon and forms an important collateral link with the middle colic artery via the marginal artery of Drummond. If the IMA trunk is ligated, this marginal arcade can offset ischemia, but the splenic flexure region is a classic “watershed” zone at the border between superior mesenteric artery (SMA) and IMA perfusion, and its blood supply is notoriously variable. In about 30% of elderly patients, IMA flow stops at the splenic flexure, leaving the distal colon dependent on SMA-derived collaterals (4). A complete marginal arterial arch exists in >95 % of individuals, whereas a robust arc of Riolan (a large anastomosis between the middle and left colic arteries) is rare, occurring in only 1%–2%  (4).

These variations highlight the value of preoperative assessment: if the LCA is absent or the marginal arcade is discontinuous, high tie ligation could markedly raise the risk of distal colonic ischemia.

### 2.2 Physiology of the LCA and colonic perfusion

The LCA is the primary blood source for the distal descending and proximal sigmoid colon; its ascending branch also feeds the splenic flexure via the marginal artery. Preserving the LCA therefore maintains direct inflow to the residual colon and anastomosis after low anterior resection. High-tie ligation, in contrast, sacrifices proximal IMA flow so that the entire distal supply must rely on SMA-mediated collaterals. Experimental work supports the physiological impact: using laser Doppler, Seike et al. showed that perfusion at the sigmoid stump was significantly lower after high tie than after low tie (8).

Adequate perfusion is critical for anastomotic healing, so from a physiological standpoint, keeping the LCA should lower the risk of ischemic leaks. Nevertheless, in patients with diffuse atherosclerosis, poor LCA flow, or an already well-developed collateral arcade, the hemodynamic hit from a high tie may be trivial. Preoperative CTA/MRA to gauge colonic perfusion is therefore especially useful in older patients or those with vascular disease, helping to decide whether LCA preservation is truly necessary (4).

### 2.3 Lymphatic-drainage anatomy

Rectal lymphatic outflow tracks closely with IMA branching. Lymph from the upper rectum ascends along the superior rectal artery to the “central” nodes at the IMA root, whereas mid- and lower-rectal drainage can also reach the internal-iliac chain laterally. High-tie surgery removes the IMA root *en bloc* with its surrounding fatty tissue, achieving a complete D3 central-node dissection. Although the incidence of “apical” node metastasis at the IMA root is low (generally < 5 %), its presence signals worse prognosis and a higher risk of distant spread (5). Kim et al. reported that metastasis to nodes alongside the IMA trunk correlated with advanced tumor stage and poorer survival, underlining the value of root dissection (5).

Low-tie ligation need not forgo central clearance. An experienced surgeon can preserve the LCA while still meticulously excising nodes around the IMA origin—achieving oncological radicality comparable to a high tie (“low tie plus high dissection”)  (9). Understanding these lymphatic routes therefore helps tailor the operation: the aim is to balance oncological safety with preservation of arterial inflow whenever feasible.

## 3 Surgical approaches

### 3.1 Open procedures and minimally invasive techniques

Whether the operation is open, laparoscopic, or robot-assisted, management of the IMA is a key step in anterior resection. In an open field, the IMA and LCA are readily identified, so the surgeon can choose a high or low tie as needed. Laparoscopic magnification delineates the IMA root clearly, and some surgeons prefer a straightforward high tie beside the aorta because exposure is intuitive and rapid (5). Others report that, with adequate skill, the LCA can be safely preserved laparoscopically, provided the bifurcation between the LCA and superior rectal artery is meticulously dissected, a task that demands greater dexterity (6). Robotic platforms, with articulated instruments and high-definition 3-D vision, may further facilitate precise branch dissection and nerve protection, making LCA-preserving low ties more feasible. In general, the choice of approach hinges on surgeon experience and patient anatomy: minimally invasive surgery can achieve oncological clearance and vascular control equivalent to open procedures, but only if the vessels are fully exposed and correctly identified, avoiding inadvertent ligation of the LCA or injury to critical structures (9).

### 3.2 Intraoperative perfusion assessment

When the LCA is to be preserved, surgeons must confirm that the remaining colon is well perfused. Traditional indicators such as palpable arterial pulsation, bowel color and warmth, or bleeding at the cut edge are subjective. Fluorescence imaging has brought a more objective alternative: after intravenous indocyanine green (ICG), near-infrared cameras display real-time perfusion on the serosal surface, allowing direct visualization of distal flow (10). If fluorescence is delayed or patchy after LCA preservation, the surgeon can re-resect to a better-perfused level, or, if necessary, abandon LCA preservation altogether to secure an adequately vascularized anastomosis. A large multicenter RCT tested whether ICG imaging reduces anastomotic leaks; overall 90-day leak rates did not differ, but subgroup analysis suggested fewer clinically significant leaks in low rectal anastomoses  (10). Thus, ICG is a helpful adjunct that sharpens intraoperative decisions, though its impact depends on timely use and disciplined responses to abnormal perfusion. Additional methods include handheld Doppler assessment of marginal flow or transient clamping of the LCA while observing bowel color. Among them, ICG fluorescence is the most intuitive and increasingly routine, guiding instant decisions in LCA-preserving surgery and avoiding occult ischemia.

### 3.3 High-tie versus low-tie ligation

High tie simplifies central lymph-node dissection and yields a slightly longer colon for a low anastomosis, but after LCA division the splenic flexure must be fully mobilized to eliminate undue tension. Low tie maintains continuous blood supply to the descending colon, theoretically aiding anastomotic healing and functional recovery. Even so, thorough nodal clearance around the IMA remains essential; surgeons should not compromise oncological radicality for vessel preservation. Likewise, they must confirm that the residual colon is long enough and, when in doubt, mobilize the splenic flexure in advance or adopt other measures. Each strategy has strengths and drawbacks; the optimal choice depends on patient anatomy, tumor factors, and real-time perfusion findings (Table 1).

**Table 1:** Key technical differences between high-tie and low-tie ligation of the inferior mesenteric artery.

Aspect	High-tie ligation (LCA sacrificed)	Low-tie ligation (LCA preserved)
Ligation site	IMA ligated at its origin from the aorta	IMA ligated distal to the LCA branch; LCA retained
Anastomotic perfusion	Depends solely on SMA collaterals; distal flow reduced	Direct arterial inflow maintained; richer perfusion at anastomosis
Mobilizable colon length	Longer proximal colon; easier tension-free anastomosis	Slightly shorter; may need full splenic-flexure mobilization
Technical complexity	Simple: identify and ligate IMA root	More demanding: dissect and identify LCA, superior rectal artery, etc.
Lymphatic clearance	Includes central nodes at IMA root (D3 dissection)	Typically clears up to superior rectal artery; IMA root nodes can be added
Autonomic nerve preservation	Proximity to sympathetic plexus; higher risk of injury	Farther from plexus; lower risk of pelvic autonomic damage

### 3.4 Common complications and their management

Anastomotic leakage remains the chief postoperative complication after LCA-preserving surgery and warrants vigilant prevention (see later section on peri-operative outcomes). Risk can be curtailed by ensuring robust perfusion, constructing a tension-free anastomosis, and creating a protective stoma when indicated. High ties, performed near the aorta and the sympathetic plexus, carry a risk of bleeding or postoperative autonomic dysfunction; blunt dissection close to the arterial wall and careful nerve preservation are therefore mandatory. During low ties, the small LCA branches must be protected; if injured, they should be promptly ligated to forestall uncontrollable bleeding from traction tears. Another potential issue is sigmoid-rectal anastomosis torsion or compromised perfusion caused by mesenteric stump traction; partial mesenteric division or bowel re-orientation can relieve this. Ultimately, meticulous, oncologically sound technique is the cornerstone of complication avoidance in either ligation mode. Should problems arise, such as leaks, urinary dysfunction and so on, early intervention, including reoperation or a diverting stoma when necessary, minimizes patient risk and improves outcomes.

## 4 Indications and contraindications

### 4.1 Tumor stage

For early rectal cancer, specifically stage I and selected stage II cases with no radiologic evidence of nodal metastasis and negative high-level nodes, surgeons generally favor a low tie that preserves the LCA to maximize blood supply and functional protection, because the probability of apical IMA node metastasis is extremely low and the additional clearance gained from a high tie is small (5). In contrast, for locally advanced disease, some surgeons prefer a high tie to remove all potentially metastatic nodes and reduce residual disease. Others argue that, even with LCA preservation, an adequate D3 lymphadenectomy can achieve similar oncological radicality (9). Current meta-analyses show no significant survival difference between high and low ties in stage III patients  (9, 11); thus, even in advanced cases, LCA preservation remains acceptable provided nodal clearance is thorough. In principle, an advanced stage is not an absolute contraindication to LCA preservation, but the surgeon must carefully map any bulky nodes and be sure they can be completely removed.

### 4.2 Tumor location and depth

Upper rectal tumors usually require IMA management during low anterior resection. When the lesion lies in the sigmoid colon or high rectum, well above the LCA, preserving the LCA does not compromise proximal vascular control or a safe proximal margin and is therefore more feasible. By contrast, for mid to low rectal cancers, especially ultra low resections, maximum colonic length is needed for a tension free anastomosis, and in this context a high tie may help by adding mesenteric slack (6). Thus, in extremely low rectal cancers where marginal length is clearly inadequate, a high tie combined with complete splenic flexure mobilization may be warranted. If the tumor directly invades the IMA trunk (a rare scenario) or if tumor thrombus or fused nodes encase the LCA, a high tie is required for radical excision. In general, the lower the tumor and the more demanding the operation, the more carefully surgeons must balance perfusion against colonic length and make decisions on a case by case basis.

### 4.3 Vascular anatomy

If pre-operative imaging shows congenital absence of the LCA, a diminutive or occluded LCA, or diffuse arteriosclerosis, the artery contributes little to distal perfusion and preserving it serves no practical purpose; a high tie can remove disease without increasing leak risk (4). Conversely, if CTA reveals an interrupted marginal artery and a watershed at the splenic flexure, indicating that the descending colon relies mainly on the LCA, high tie would markedly raise the ischemic-anastomosis risk and LCA preservation is preferable. In some patients the IMA trunk is very short and the LCA originates close to the aorta (“low-origin” variant), making a low tie technically hazardous; forcing a low tie in that anatomy risks LCA injury or incomplete central node clearance. When low-tie dissection is anatomically difficult, a high tie can be the safer option (6).

### 4.4 Neoadjuvant therapy

Rectal-cancer patients who receive neoadjuvant chemoradiation often have tumor shrinkage but increased local edema and fibrosis, and radiation may impair regional vessels. Their leak risk is already higher than in non-irradiated patients, so preserving the LCA to boost local perfusion is even more desirable. On the other hand, post-radiation nodal assessment is unreliable, and occult microscopic disease may persist centrally. Experience suggests that neoadjuvant therapy does not by itself change tie-level criteria, but intra-operative perfusion checks with ICG are especially useful, and fibrotic tissue must be handled cautiously with maximal vessel protection. Prior abdominal surgery or other factors that alter mesenteric collaterals likewise mandate intra-operative flexibility. When in doubt, a protective proximal stoma on top of a low tie can buffer against potential hypoperfusion at the anastomosis.

## 5 Oncologic outcomes

Comparing the long-term oncologic performance of high-tie ligation with LCA-preserving surgery after curative resection is central to weighing the two strategies. Key endpoints include overall survival (OS), disease-free survival (DFS), local-recurrence rate, and the pathological quality of the specimen (see Table 2).

**Table 2:** Comparison of oncologic and functional/peri-operative outcomes: high-tie vs. LCA-preserving (low-tie) ligation.

Outcome metric	High-tie ligation/high-tie group	LCA-preserving low tie/low-tie group
Oncologic outcomes		
5-year Overall Survival (OS)	≈ 88%–91%	≈ 88%–91%
5-year Disease-Free Survival (DFS)	≈ 82%–84%	≈ 82%–84%
Local recurrence rate	≈ 5%–8%	≈ 5%–8%
Average harvested lymph nodes	≈ 21–23	≈ 18–20
Central (apical) node positivity	Comparable (no significant difference)	Comparable (no significant difference)
Distal/circumferential margins (R0)	No difference in R0 rate	No difference in R0 rate
TME specimen quality	Comparable completeness	Comparable completeness
Mean 5-year Overall Survival (OS)	79.8%	80.3%
Mean 5-year Disease-Free Survival (DFS)	75.8%	75.9%
Functional and peri-operative outcomes		
Anastomotic leak rate	≈ 10% (lower when a diverting stoma is used)	≈ 5%–8%; significantly lower than high-tie
Post-operative urinary dysfunction	May occur; some cases of retention or dysuria	Slightly lower incidence; retention less common
Post-operative sexual dysfunction	Risk of male erectile/ejaculatory impairment	Better preservation of sexual function (≈ 80%–90% of normal)
Bowel function (LARS)	Marked dysfunction within 3 months; some patients still have moderate/severe LARS at 1 year	Faster recovery; most patients show improved LARS scores by 1 year
Mean operative time	Slightly shorter	Slightly longer (5–15 min)
Time to first flatus	≈ 2–3 days post-op	Within ≈2 days (earlier)
Mean length of stay	≈ 8–10 days	≈ 7–9 days (slightly shorter)

### 5.1 Overall survival and recurrence

**Figure 1:**
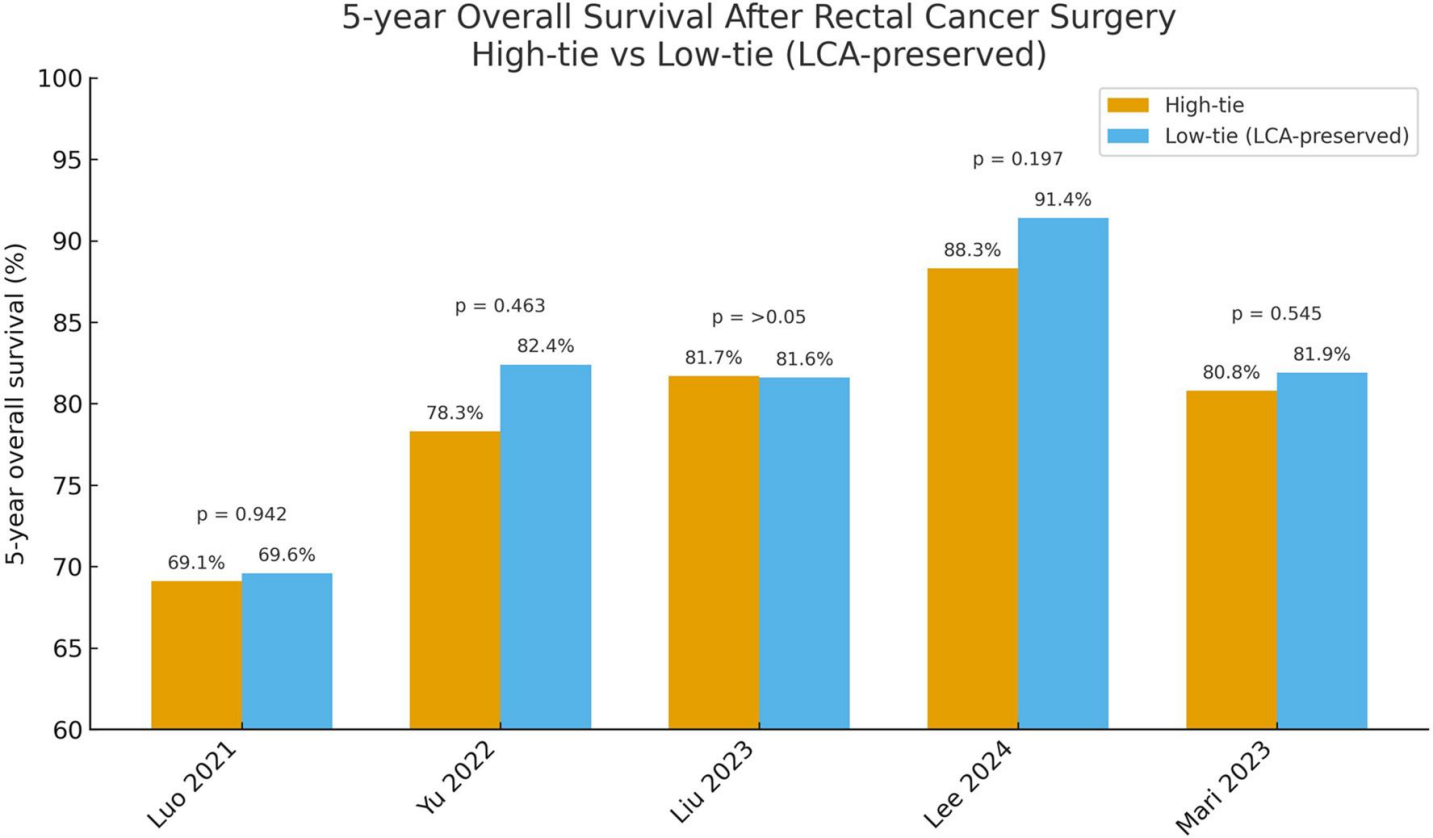
Comparison of 5-year overall survival between high-tie and low-tie (LCA-preserving) ligation of the inferior mesenteric artery.

**Figure 2:**
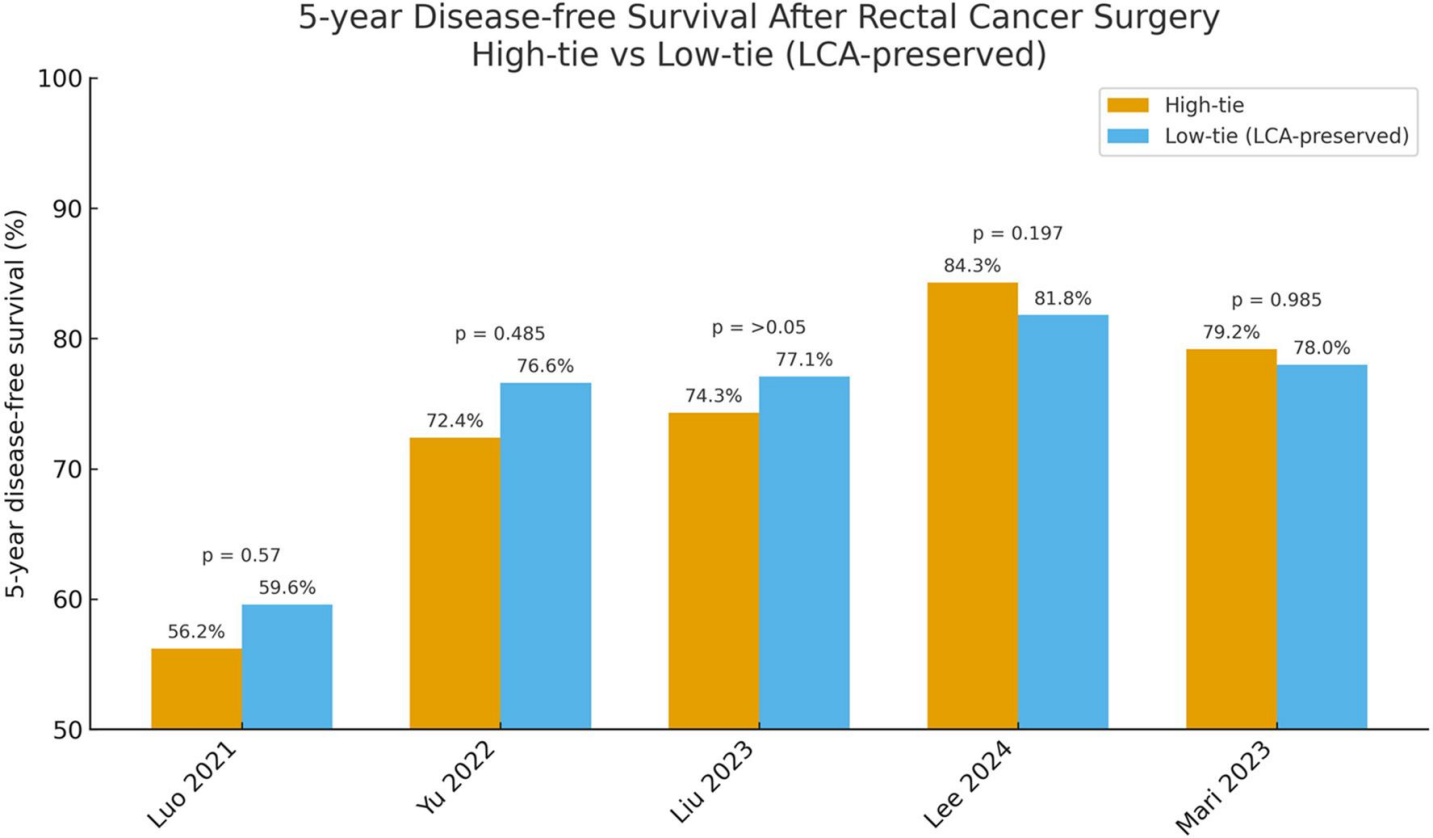
Comparison of 5-year disease-free survival between high-tie and low-tie (LCA-preserving) ligation of the inferior mesenteric artery.

A substantial body of evidence shows that high-tie ligation does not significantly improve long-term survival in rectal-cancer patients. Most randomized trials and retrospective studies report virtually identical 5-year OS for both approaches (11–15). In a Chinese multicenter retrospective cohort, the 5-year OS was 69.6 % in the LCA-preservation group vs. 69.1 % in the high-tie group, a nonsignificant difference (12). The recently published five-year follow-up of the HIGHLOW randomized controlled trial confirmed these findings: 5-year survival was 88.3 % after low tie and 91.4 % after high tie (*P* = 0.20), and DFS was likewise indistinguishable (11). A meta-analysis by Yang et al. pooling eight studies reached the same conclusion: the two techniques yielded essentially equivalent 5-year survival, with no tilt in risk ratios (8). As for local recurrence, no evidence indicates that high tie lowers the rate. Most reports show minimal differences between groups, with recurrence risks typically around 5%–10%, and distant metastasis rates are likewise similar (12, 16). It is worth emphasizing that a patient's prognosis is driven chiefly by tumor biology (stage, histology) and the quality of total mesorectal excision, not by the IMA ligation level *per se*. (See Figures 1, 2).

### 5.2 Lymph-node harvest and pathological metrics

Because high-tie surgery includes clearance at the IMA root, it might theoretically yield a higher total node count. Some studies do find a modest increase: one report noted an average of 23 nodes retrieved with high tie vs. 20 with LCA preservation (*P* < 0.01)  (12). Yet both numbers comfortably exceed the minimum of 12 nodes required for accurate staging, so the difference carries little practical weight. The extra nodes harvested by high tie are largely a few “apical” nodes at the IMA root, whose positivity rate is very low and seldom alters staging. Meta-analyses confirm that total node counts differ only marginally and do not affect N-category assignment (9, 16). More importantly, low-tie ligation does not hinder thorough regional nodal clearance. Studies comparing central-node positivity between techniques find no significant disparity (15). Pathologic margins and TME quality are likewise unaffected: the HIGHLOW trial reported no differences in distal or circumferential margins (R0 rate) or Quirke-graded mesorectal integrity (17). Matsuda et al.'s randomized study also showed comparable TME quality and margin status regardless of ligation height (18). In short, when the operation is performed to standard, preserving the LCA neither compromises radicality nor leaves key lymph nodes behind.

## 6 Peri-operative and functional outcomes

### 6.1 Anastomotic leak

Most investigators agree that preserving the LCA lowers the risk of anastomotic leak after low anterior resection, chiefly because the artery delivers richer perfusion to the anastomosis. Several retrospective studies support this view: in a propensity-matched series, clinical leaks occurred in only 3.3 % of patients whose LCA was preserved vs. 13.3 % in those without preservation (*P* = 0.048)  (12). A 2018 meta-analysis that pooled 11 studies (3,000 patients) found that foregoing the LCA increased leak risk by roughly 22 % (OR: 1.28, *P* < 0.05)  (16). A recent *Frontiers* review combining data from four RCTs and 13 cohort studies likewise concluded that low-tie ligation combined with high-level nodal dissection significantly reduced overall leak rates (*P* < 0.001)  (9). Experimental work in 40 rectal-cancer patients showed that clamping the IMA intra-operatively markedly decreased flow in the marginal arteries near the anastomosis, whereas keeping the LCA mitigated this perfusion deficit and lowered leak risk (19).

It must be noted, however, that a few RCTs have failed to show a significant difference. In a small Japanese trial by Fujii et al., clinical leak rates were 7.4 % with high tie and 6.0 % with low tie, a nonsignificant gap (20). Early results of the HIGHLOW trial similarly reported leak rates of about 5–6 % in both groups when a diverting stoma was used (*P* > 0.5) (17). Such discordance may reflect (i) selection bias in retrospective studies, where surgeons tend to preserve the LCA in high-risk cases with poor distal perfusion, and (ii) the limited sample sizes and routine defunctioning in RCTs, which lower absolute leak rates and reduce statistical power. Taken together, the evidence still favors LCA preservation, particularly when a protective stoma is not employed.

### 6.2 Urinary and sexual function

Radical rectal surgery often jeopardizes autonomic nerves, leading to male erectile/ejaculatory dysfunction and urinary disorders in both sexes. High-tie ligation is performed close to the peri-aortic sympathetic plexus (the inferior mesenteric and superior hypogastric plexuses); careless dissection can therefore impair bladder detrusor or sexual function (6). Low-tie ligation, farther from the main nerve trunks, theoretically spares more autonomic fibers. In the HIGHLOW RCT, urinary function scores three months after surgery were significantly better in the low-tie group (lower IPSS), and male sexual function recovered more favorably (17). Follow-up by Mari et al. showed that these differences persisted at one year (17). Not every study finds a clear advantage: a Korean single-center series reported similar rates of postoperative autonomic dysfunction (3%–5%, *P* > 0.05) in both groups, perhaps because all operations were performed by surgeons highly experienced in nerve preservation (6). Overall, most data support the notion that low tie is kinder to postoperative urinary and sexual function, likely because it avoids excessive traction or division around the IMA origin. Still, nerve outcomes also depend on pelvic nerve-sparing technique, tumor stage (and hence TME difficulty), and other factors; ligation level is only one piece of the puzzle.

Focusing on women, urinary and sexual dysfunction is common and may persist long-term after rectal cancer treatment—particularly following TME and (neo)adjuvant radiotherapy. Population-based studies report postoperative urinary urgency/incontinence rates up to 77%/63%, and vaginal dryness, dyspareunia, and reduced vaginal dimensions of approximately 72%, 53%, and 29%, respectively; preoperative radiotherapy is independently associated with worse functional outcomes (21, 22). Mechanistically, injury to the superior hypogastric plexus and pelvic splanchnic nerves is implicated; high ligation of the IMA may increase risk, whereas low tie/left colic artery preservation was associated with better genitourinary function at 9 months in a randomized trial (17). However, systematic reviews and meta-analyses have not reached consistent conclusions regarding differences by ligation level (23). We recommend routine use of validated instruments to capture baseline and follow-up outcomes in women, and that studies prespecify female-specific functional endpoints and stratify by neoadjuvant radiotherapy to enhance comparability and interpretability (24).

### 6.3 Bowel function and LARS

Low anterior resection syndrome (LARS) is common after sphincter-saving surgery, manifesting as frequent stools, urgency and incontinence. By maintaining the LCA, surgeons not only improve perfusion but also limit the extent of colonic denervation, helping preserve compliance and transit. In a randomized trial, Matsuda et al. assessed bowel function at three months and one year using patient questionnaires; early scores (Wexner incontinence, FIQL and self-rated difficulty) were similar, but by one year the low-tie group had clearly better recovery, with significant improvement in FIQL and Wexner scores, unlike the high-tie group (18). Recent retrospective analyses echo this trend: one report found significantly lower FISI scores at one year in LCA-preserved patients, implying better long-term continence (6). The mechanism may be that high tie removes sympathetic input for a longer proximal segment (sympathetic fibers accompany the IMA for about 5 cm), provoking dysmotility and spastic contractions and hence frequent, poorly controlled stools (10). Koda et al. showed more spastic colonic waves in high-tie patients, correlating with serious bowel dysfunction (10). Preserving the LCA, and thus some neural supply, appears to hasten functional recovery, particularly during the first postoperative year.

Additional support for the link between vascular and autonomic preservation and postoperative bowel function comes from the work of Dulskas and colleagues (25–27). In a series of prospective studies, they reported high rates of LARS and defecatory disorders after low anterior resection and showed that impaired rectal reservoir function, anastomotic ischaemia and pelvic autonomic nerve injury were key determinants of poor functional recovery rather than tumour stage alone. Dulskas et al. also emphasised that more extensive mesenteric devascularisation and denervation were associated with worse Wexner, FIQL and LARS scores at long-term follow-up. Although these studies did not specifically randomise patients by ligation height, their findings indirectly support the concept that preserving the LCA, and thereby maintaining better perfusion and limiting the length of denervated colon, may mitigate severe LARS and improve long-term bowel function.

### 6.4 Other peri-operative metrics

Beyond the major complications and functions above, the two techniques differ slightly in general peri-operative recovery. LCA preservation usually entails more meticulous vascular dissection, adding a small amount of operative time. One retrospective study recorded a mean increase of 13 min (225 vs. 212 min, *P* = 0.039) for low tie (12); meta-analyses report (28) a weighted mean extra time of about +10 min, a negligible impact on risk. Conversely, postoperative recovery tends to favor LCA preservation: better anastomotic perfusion promotes healing, so bowel function returns sooner and hospital stay may be shorter. Some reports show the first flatus occurring 0.3 days earlier (*P* < 0.01) and length of stay reduced by 0.6 days after low tie (10). In a retrospective series of 70 laparoscopic resections, LCA preservation led to significantly earlier flatus and discharge, smaller declines in gastrointestinal hormones, and a lower overall complication rate (5.88 % vs. 27.78 %, *P* < 0.05) (29, 30). An analysis of 888 Japanese patients likewise found fewer leaks in the preservation group, smoothing postoperative recovery (30). (See Table 2).

## 7 Blood-supply assessment and imaging

### 7.1 Pre-operative CTA/MRA

As noted above, IMA/LCA anatomy and collateral circulation vary considerably among patients. Multidetector CT angiography (CTA) now makes it possible to map these differences before surgery. CTA clearly delineates the IMA origin, the LCA bifurcation, and the continuity of the marginal artery. Murono et al. showed that 3-D CTA non-invasively provides the IMA length and branching pattern, allowing surgeons to anticipate the ideal ligation level and extent of nodal clearance (3). In elderly patients or in those suspected of peripheral vascular disease, CTA can detect high-risk features such as LCA stenosis or occlusion and poorly developed collaterals, alerting the surgeon to preserve the artery intra-operatively to avoid ischemia.  (4) Magnetic-resonance angiography (MRA) is a viable alternative for those without contraindications to contrast, though its spatial resolution is slightly lower. Overall, pre-operative vascular imaging personalizes the surgical plan: absent LCA on CTA points to a straightforward high tie, whereas a splenic-flexure watershed with no collateral arc argues for LCA preservation and full splenic-flexure mobilization. With digital medicine advancing, CTA-based three-dimensional reconstructions can even be explored in virtual-reality simulations, helping surgeons rehearse vascular control and enhancing precision and safety.

### 7.2 ICG fluorescence perfusion imaging

Intra-operative indocyanine-green (ICG) fluorescence, discussed earlier, merits emphasis for its clinical promise. By displaying real-time distal colonic perfusion, ICG offers an objective check that LCA preservation is giving the anastomosis sufficient blood. Single-center studies show that ICG-guided margin adjustment lowers leak rates in high-risk patients (10). ICG fluorescence is becoming a staple of ERAS protocols in colorectal surgery, helping surgeons confirm anastomotic safety when the LCA is spared.

Some cutting-edge research goes further, applying artificial-intelligence (AI) algorithms to quantify ICG videos, aiming to improve consistency and accuracy in perfusion assessment. One report extracted brightness-histogram features from ICG frames and trained a neural network that classified perfusion as adequate or poor with >99 % accuracy (31). Integrated into laparoscopic systems, such real-time AI analysis could offer immediate, objective feedback, reducing reliance on subjective judgment. Wider adoption would boost the safety of LCA-preserving procedures and benefit more patients.

**Figure 3:**
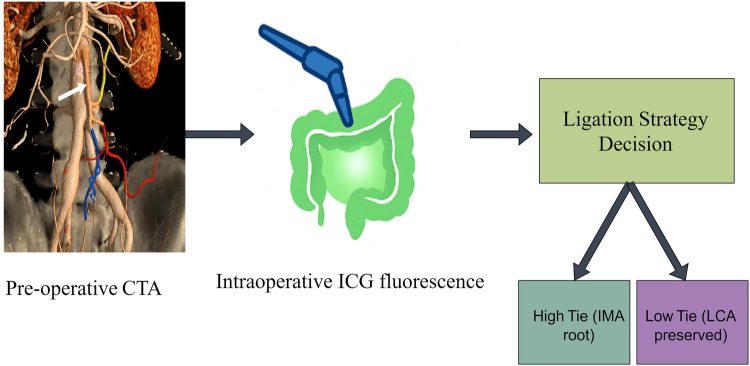
Process for evaluating the location of vascular ligation.

Others have begun using pre-operative AI models to predict which patients are good LCA-preservation candidates. Deep-learning techniques that merge CTA images with clinical data can forecast the risk of ischemic complications after a high tie, allowing individualized plans in advance (32). These explorations are preliminary, but they point toward a future in which AI-augmented imaging delivers end-to-end perfusion management—precise pre-operative assessment and real-time intra-operative monitoring. In such a framework, the decision to preserve the LCA becomes more scientific and objective, and surgeons can choose the optimal strategy with greater confidence (see Figure 3).

Despite these promising proof-of-concept studies, AI-assisted perfusion assessment and decision-support systems are still at an early stage. Most existing models are trained on small, single-centre datasets and lack external validation; overfitting, domain shift and limited interpretability remain major concerns. In addition, regulatory approval pathways, medico-legal accountability, data-privacy issues and the need for seamless integration into existing laparoscopic platforms all pose non-trivial hurdles. As such, AI tools should currently be regarded as adjuncts to expert surgical judgement rather than replacements, and future multicentre trials with prespecified endpoints and transparent reporting will be essential before routine adoption can be recommended.

### 7.3 Other assessment techniques

Beyond CTA and ICG, a few adjunct tools aid perfusion evaluation and operative planning: endoscopic perfusion grading (mucosal color), intra-operative laser-Doppler probes measuring bowel wall flow, and optical-coherence tomography (OCT) for microcirculation, among others (33). Most remain investigational. For patients with especially poor collaterals, interventional radiology can perform selective angiography to map marginal-artery perfusion (4). If the descending colon relies entirely on the LCA, the artery should be preserved; if its territory is minimal, high tie is safe. A staged ligation concept, in which the IMA is embolized several days before surgery to promote collateral development, has been reported for cases where a high tie is needed but distal ischemia is feared, although validation in large scale studies is lacking.

As concepts evolve, “perfusion optimization” has become a central goal in colorectal cancer surgery. With diverse imaging and technical adjuncts, surgeons can gauge blood flow more accurately and make wiser choices between high and low tie.

## 8 Comparative studies and systematic reviews

### 8.1 Randomized controlled trials (RCTs)

Although few in number, several prospective trials have compared ligation levels. The landmark Italian HIGHLOW trial randomized 240 patients undergoing laparoscopic anterior resection to high or low tie (17). Early reports showed no difference in node harvest or R0 resection rate, but significantly better urinary and sexual scores at three months in the low-tie arm, with similar leak rates. Five-year follow-up, published in 2023, confirmed virtually identical OS and DFS, reinforcing that low tie does not compromise survival (11). A Japanese RCT by Fujii et al. aimed primarily at leaks but terminated early after enrolling only 59 patients; it found no statistical difference in leaks or complications (20). Another Japanese RCT by Matsuda et al. focused on bowel function; while early results were similar, the low-tie group showed better long-term recovery (18). In short, existing RCTs are small and heterogeneous in endpoints, yet none demonstrate a clear advantage for high tie, and functional metrics favor low tie, with the most striking difference seen in the HIGHLOW trial. Larger, multicenter RCTs are still needed to strengthen the evidence base.

### 8.2 Retrospective cohort studies

Given RCT limitations, much insight comes from large retrospective series. A Korean center's analysis of 614 anterior resections found no OS or DFS difference between low-tie + D3 dissection and high tie, and similar leak and urinary-complication rates (9). Multiple Chinese single-center studies likewise show that LCA preservation does not impair oncologic results while lowering leak risk (12). Propensity-score matching often balances baseline differences: for instance, a matched analysis from Daping Hospital (Chongqing) confirmed reduced leaks with LCA preservation and no difference in node harvest or three-year survival (12). Still, retrospective data are prone to selection bias, as surgeons may choose low tie for frailer patients, and to varying outcome definitions and center-specific practices, so results must be read cautiously. Overall, however, the trend is consistent: long-term tumor control is equivalent, whereas peri-operative outcomes often favor LCA preservation.

### 8.3 Meta-analyses and systematic reviews

To synthesize scattered studies, several meta-analyses have emerged. Fan et al. (2018) combined 19 studies and found no OS or recurrence difference, but a significantly lower leak rate with LCA preservation (16). Hajibandeh et al. (2020) pooled only RCTs and performed trial-sequential analysis (TSA), again seeing no survival gap and concluding that further trials are unlikely to reveal a > 5 % high-tie advantage (8). A 2021 review focused on leaks and, while finding no statistically significant difference, noted that limited RCT numbers prevent firm conclusions (28). Another 2021 meta-analysis compared “low tie + high-level D3 clearance” directly with high tie, showing markedly fewer leaks with the former and no oncologic disadvantage, recommending it for experienced surgeons (9). A recent synthesis of 15 studies (5,054 patients) likewise reported lower leak rates with LCA preservation (OR = 1.03, 95 % CI 0.83–1.27, *P* < 0.0001) and no differences in operative time, blood loss, node count, overall complications, systemic or local recurrence, five-year OS, or DFS (34). More recent Western reviews have reached similar conclusions (35–38). Brillantino et al. provided a comprehensive narrative synthesis of IMA ligation strategies in *Cancers* (2024), underscoring the absence of a survival advantage for high tie and supporting selective use of LCA preservation in experienced hands (36). Likewise, Negoi's 2025 umbrella review in *Life* integrated current surgical guidelines and concluded that ligation height should be individualised, with LCA preservation generally preferred when D3 lymphadenectomy and adequate perfusion can both be secured (38). These Western data help to balance the predominantly East-Asian evidence base and strengthen the generalisability of LCA-preserving strategies to non-Asian populations.

### 8.4 Evidence quality and guideline grading

Current evidence for ligation level sits at a moderate tier of the evidence hierarchy. Western guidelines such as the NCCN do not mandate a specific ligation height, largely because conclusive high-level data are lacking. Japanese JSCCR guidance, based on level-II evidence, recommends a high-tie D3 dissection for advanced disease but acknowledges low tie in selected circumstances (5). Most RCTs emphasize functional or short-term endpoints; large trials powered for long-term oncologic outcomes remain absent. Thus, the evidence for LCA preservation is roughly grade 2A: multicenter cohort data and small RCTs support it, but decisive phase-III evidence is missing. Many authors call for large, prospective, multicenter randomization, though others argue such trials may be impractical or unethical if surgeon preference already leans one way. Practically, robust propensity-score analyses, prospective registries, and granular subgroup studies may be more feasible, helping reveal which patients benefit most and which benefit least from LCA preservation. In other words, rather than seek a “one-size-fits-all” verdict, future research should refine individualized decision-making, which represents the next frontier for this debate.

## 9 Controversies and future directions

### 9.1 Does high-tie ligation confer long-term benefit in a specific subgroup?

Although high-tie ligation does not improve survival overall, some authors argue it could help the subset of patients with apical IMA-node metastasis by removing additional tumour load and thus enhancing prognosis. One study showed that patients with positive nodes above the LCA had a much worse 5-year DFS than those without involvement (31.9 % vs. 69.4 %)  (39). On this basis, some surgeons speculate that a high tie, combined with para-aortic dissection, might eradicate occult micrometastases in node-positive cases and improve outcomes (40). Others counter that systemic relapse risk is already high in these patients, so more aggressive local clearance offers little gain  (41). To date, no randomised trial has targeted the “central-node-positive” subgroup. Large cohort analyses or trials designed for this high-risk population are needed to clarify whether high tie delivers a survival benefit in any defined subgroup.

Another important limitation is regional anatomical variability. Many of the largest series favouring LCA preservation originate from East-Asian centres, where patients tend to have lower BMI, slimmer mesentery and somewhat different IMA branching patterns compared with Western populations (42, 43). These factors may make vascular dissection and nerve preservation technically easier and may not fully reflect the challenges encountered in obese patients or in those with extensive atherosclerosis, who are more common in Europe and North America. Future prospective studies should therefore deliberately include diverse populations and report outcomes stratified by region, BMI and vascular risk profile to clarify how generalisable current findings truly are.

### 9.2 Situations where high tie remains appropriate

While the preponderance of evidence favours LCA-preserving low-tie ligation for most patients, there remain clearly defined scenarios in which high tie may still be appropriate or even preferable. First, in the presence of bulky apical or para-aortic nodes encasing the IMA root, an *en bloc* high-tie dissection may be the only way to achieve an R0 resection and complete central lymphadenectomy (44). Second, in patients with complex vascular anatomy, such as a very short IMA trunk, a low-origin LCA branching within a few millimetres of the aorta, or dense post-radiation fibrosis, attempting a low tie can be hazardous and may increase the risk of LCA injury or incomplete nodal clearance (45). Third, when pre-operative imaging shows an absent or severely stenotic LCA and a well-developed marginal arcade from the SMA, preserving the LCA is unlikely to improve perfusion, whereas a high tie can simplify the procedure without materially worsening anastomotic blood supply (46). These counter-examples highlight that LCA preservation should not be viewed as dogma; instead, ligation height must be individualised according to oncologic risk, vascular anatomy and technical feasibility (Table 3).

**Table 3:** Clinical scenarios favouring LCA preservation vs. high-tie ligation.

Clinical scenario	Suggested ligation strategy	Rationale/risk–benefit consideration
Early-stage sigmoid or upper rectal cancer without bulky apical nodes	Prefer low tie with LCA preservation	Central lymph node burden is low; preserving the LCA optimises anastomotic perfusion and may reduce anastomotic leakage and severe LARS, without clear oncologic benefit from routine high tie.
Low/mid rectal cancer after TME with good distal perfusion on imaging or ICG	Prefer low tie with LCA preservation	Adequate mesenteric shortening can still be achieved; maintaining LCA inflow helps preserve marginal arcade perfusion to the neorectum and avoids additional autonomic denervation.
Markedly attenuated marginal artery at the splenic flexure	Strongly favour low tie with LCA preservation	Anastomotic segment relies heavily on LCA inflow; high-tie ligation may precipitate colonic ischaemia and increase risk of leakage, strictures and poor functional recovery.
Frail, elderly or high-risk patient with limited physiological reserve	Prefer low tie with LCA preservation	Maximising blood supply to the anastomosis is prioritised; any increase in leak, reoperation or sepsis related to ischaemia may be poorly tolerated in this vulnerable group.
Locally advanced tumour with bulky apical/para-aortic nodes around the IMA root	Consider high tie IMA ligation	En bloc high-tie dissection facilitates complete central node clearance and may be necessary to achieve R0 resection; oncologic safety outweighs the potential perfusion benefit of LCA preservation in this setting.
Complex vascular anatomy with very short IMA trunk or LCA branching close to aorta	High tie often safer and more practical	Attempting a low tie may risk inadvertent LCA injury, bleeding or nerve damage; a controlled high tie may provide a clearer field and more reproducible lymphadenectomy in unfavourable anatomy.
Absent or severely stenotic LCA with robust SMA-derived collaterals	High tie acceptable	Preserving a functionally compromised LCA is unlikely to improve perfusion; high tie simplifies the procedure and is unlikely to worsen anastomotic blood supply when proximal collaterals are well developed.
Dense post-radiation fibrosis around the IMA root	Individualised; low tie desirable if safely feasible	Radiation fibrosis makes precise LCA-preserving dissection difficult; LCA preservation could mitigate ischaemia in irradiated bowel, but not at the expense of uncontrolled bleeding or nerve injury—strategy should depend on intra-operative findings and perfusion assessment.
Need for maximal bowel length	Prefer low tie with LCA preservation	Preserving the LCA allows better perfusion of a long colonic segment brought down to the pelvis, potentially improving healing and long-term function compared with a fully devascularised conduit.
Emergency setting with haemodynamic instability or gross contamination	High tie may be reasonable	Surgical goals shift toward rapid, safe source control; a straightforward high tie can shorten operative time and simplify dissection when detailed vascular preservation is not feasible or safe.

### 9.3 Tailored ligation strategies for vascular high-risk subgroups

We recommend that future research deliberately include vascular high-risk populations, especially patients with diabetes or diffuse atherosclerosis, because compromised microvascular perfusion may magnify the impact of inferior mesenteric artery ligation height on anastomotic blood flow and long-term outcomes. A feasible roadmap would encompass: (i) objective patient stratification using pre-operative CTA or MRA combined with AI-based perfusion scoring; (ii) intra-operative use of ICG fluorescence or comparable real-time blood-flow monitoring to adjust the choice between high-tie and low-tie dynamically on an individual basis; (iii) multi-centre prospective studies that pre-specify these comorbidities as stratification factors when comparing ligation strategies; and (iv) the creation of an international registry that continuously aggregates real-world data from heterogeneous populations. Coupled with machine-learning decision tools that integrate imaging, intra-operative haemodynamics and baseline vascular risk, this framework is expected to generate robust, personalised clinical guidelines clarifying whether high-tie ligation provides a long-term benefit and identifying the specific patients in whom it is most advantageous.

### 9.4 Strategy in the elderly and comorbid

Elderly or multimorbid patients tolerate anastomotic leak poorly, which makes LCA preservation especially attractive. These patients also often have atherosclerosis and weak collaterals, so safeguarding perfusion is critical. On the other hand, preserving the LCA can lengthen and complicate the operation—potentially problematic for frail patients. A future research priority is to confirm the value of LCA preservation in the elderly. If robust evidence shows it clearly reduces complications without raising operative risk, guidelines could issue age-specific recommendations favouring low-tie procedures.

### 9.5 Impact in the era of routine neoadjuvant therapy

Most mid- and low-rectal cancers now receive pre-operative chemoradiation, changing both the operative field and tumour biology. The debate is two-fold: does tumour downsizing let surgeons preserve the LCA more liberally, or does radiogenic fibrosis make a higher tie necessary for thorough clearance? In a propensity-matched study of 1,296 patients, neoadjuvant therapy reduced local recurrence (4.1 % vs. 10.3 %, *P* = 0.004) but not distant metastasis (28.2 % vs. 27.9 %, *P* = 0.924) (47). The lung remained the most common site of spread, followed by the liver, regardless of neoadjuvant status (47). Timing also differed: local and distant failures occurred later after neoadjuvant therapy, with distant spread peaking in year 3 rather than year 2  (47). No study has yet compared ligation levels specifically in neoadjuvant-treated patients. Future analyses should dissect this subgroup to refine operative strategy.

Neoadjuvant chemoradiotherapy also complicates the discussion of ligation height. Radiation-induced fibrosis and endarteritis may compromise microvascular perfusion around the rectum and distal colon, amplifying the impact of any further reduction in inflow from high-tie ligation (48). At the same time, scarring around the IMA root can make precise LCA-preserving dissection more difficult and potentially lengthen the operation (49). Current data are insufficient to determine whether high or low tie is preferable in this setting, but it is reasonable to hypothesise that preserving the LCA when technically safe could mitigate ischaemia-related complications and improve functional recovery in irradiated patients. Prospective studies restricted to neoadjuvant-treated cohorts are needed to test this hypothesis.

### 9.6 Artificial intelligence and surgical decision-making

As noted earlier, AI could revolutionise how surgeons choose their approach. A data-driven decision-support system that integrates tumour stage, vascular anatomy, imaging features, and even genomic data could one day estimate the risk–benefit ratio of high vs. low tie for each patient (32). AI may also assist intra-operatively: real-time analysis of laparoscopic video could flag poor perfusion or excessive nerve traction and thus reduce complications. While still embryonic, such tools are progressing rapidly. With expanding datasets and computational power, AI is poised to become a key partner in complex decisions like ligation height.

### 9.7 The call for large prospective studies

Ultimately, the controversy will be settled only by high-quality prospective research. Future trials might adopt functional endpoints as primaries, with survival as secondary, and follow patients for at least five years. Rigorous quality control, including standardised technique and postoperative care, is essential to minimise inter-centre variation. The issue is not whether “high is bad” or “low is good,” but how to match each patient with the option that yields the best composite outcome. Therefore, forthcoming large-scale studies should include stratified analyses to explore interaction effects: tumour level, age, neoadjuvant status, and so on. Only by answering these questions can personalised surgery become reality.

## 10 Conclusion

Preserving the left colic artery in radical rectal-cancer surgery exemplifies the shift from “more radical” to “more optimised” surgery. A substantial body of evidence indicates that, when indications are respected and nodal clearance is meticulous, LCA preservation does not compromise oncologic radicality: long-term survival and recurrence are indistinguishable from those after high-tie ligation. At the same time, LCA preservation confers peri-operative and functional benefits—lower leak rates, better postoperative urinary and bowel function, and faster recovery—improving quality of life and long-term satisfaction.

Surgical strategy should always centre on maximising patient benefit. LCA preservation is not universal: surgeons must weigh tumour stage, vascular anatomy, and perfusion status. When expertise or conditions are suboptimal, a high tie that ensures safe completion is preferable to a forced preservation that raises complication risks. Conversely, in most anterior resections without a clear high-tie indication, preserving the LCA should be the default because it meets the dual goals of oncologic safety and functional protection. On current evidence, LCA preservation is a safe, effective, and clinically valuable option for suitable rectal-cancer patients. It improves postoperative quality of life without sacrificing tumour control, aligning with modern patient-centred surgical ideals. Ongoing research and innovation will further delineate the optimal indications for each approach, aiming for a perfect balance between cure and function—and, ultimately, greater benefit for patients.
